# Reducing the impact of diabetic foot ulcers (REDUCE): study protocol for an effectiveness and cost-effectiveness randomised controlled trial with embedded process evaluation

**DOI:** 10.1136/bmjopen-2026-118771

**Published:** 2026-05-24

**Authors:** Natasha Mitchell, Kavita Vedhara, Kieran Ayling, Alex Mitchell, Christina Sheehan, Katherine Cullen, Ruth Hart, Azeezat Adenike Okanlawon, Catherine Arundel, Katherine Bradbury, Debbie Brewin, Trudie Chalder, Nicky Cullum, Colin Dayan, Grazziela Figueredo, Deborah Fitzsimmons, Catherine Hewitt, Glen Howard, Julia Lawton, Chris Metcalfe, David Torgerson, Judith Watson, Kirsty Winkley, Lucy Yardley, Frances Game

**Affiliations:** 1 Department of Health Sciences, Alcuin Research Resource Centre, Heslington, University of York, York, UK; 2 School of Psychology, Cardiff University, Cardiff, UK; 3 Centre for Academic Primary Care, University of Nottingham, Nottingham, UK; 4 Psychology, Swansea University, Swansea, UK; 5 The University of Edinburgh Usher Institute of Population Health Sciences and Informatics, Edinburgh, Scotland, UK; 6 Psychology, University of Southampton, Southampton, England, UK; 7 King’s College London, London, England, UK; 8 Department of Psychological Medicine, King’s College London, London, UK; 9 Division of Nursing, Midwifery and Social Work, The University of Manchester, Manchester, UK; 10 School of Medicine, Cardiff University, Cardiff, UK; 11 Lifespan and Population Health, University of Nottingham, Nottingham, UK; 12 Swansea Centre for Health Economics, Swansea University, Swansea, UK; 13 Department of Health Sciences, University of York, York, UK; 14 Health Innovation East Midlands, Nottingham, UK; 15 Bristol Medical School, University of Bristol, Bristol, UK; 16 Care in Long-Term Conditions, Nursing, Midwifery and Palliative Care, King’s College London, London, UK; 17 Psychology, University of Southampton, Southampton, UK; 18 University Hospitals of Derby and Burton NHS Foundation Trust, Derby, England, UK

**Keywords:** Diabetic foot, Randomized Controlled Trial, Community-Based Participatory Research

## Abstract

**Introduction:**

Diabetic foot ulceration represents a prevalent, persistent and resource-intensive complication of diabetes. These ulcers are slow to heal, prone to recurrence and impose a substantial burden on both patients and healthcare providers. The reducing the impact of diabetic foot ulcers (REDUCE) intervention has been designed as a multifaceted approach targeting psychological and behavioural determinants linked to diabetic foot ulcer (DFU) outcomes. Following a successful pilot trial, the REDUCE trial has been designed as a pragmatic, multicentre randomised trial to compare the effectiveness and cost-effectiveness of the REDUCE intervention plus usual care versus usual care alone in reducing recurrence in people with healed DFUs. Additionally, there is an embedded process evaluation and two sub-studies which will be carried out alongside the main trial.

**Methods and analysis:**

Adults over 18 years of age, with a recently healed DFU and two lower limbs, will be identified from around 30 specialist multidisciplinary diabetic foot clinics at participating National Health Service Trusts in the UK. Patients with active Charcot neuro-osteoarthropathy, active DFU or ulcers healed for more than 12 weeks will be excluded. We will aim to recruit 544 participants (1:1 randomisation). The primary outcome for this trial will be total ulcer-free days with limbs intact (ie, without amputation) between randomisation and the end of follow-up (18 months post-randomisation). Secondary outcomes include time to re-ulceration, total number of ulcers, amputation, quality of life (EQ-5D-5L), Patient Health Questionnaire-9, Nottingham Assessment of Functional Footcare, ICEpop capability measure for adults and resource use. As part of the process evaluation, up to 20 REDUCE intervention patient-participants will be interviewed, and the healthcare professionals delivering the intervention will also be interviewed. An assessment of intervention fidelity will also be carried out.

**Ethics and dissemination:**

Ethics approval was granted by Wales 3 Research Ethics Committee (REC reference 22/WA/0053) on 16 March 2022. The findings will be presented at relevant conferences and disseminated via peer-reviewed research publications and to relevant stakeholders.

**Trial registration number:**

ISRCTN15570706.

**STRENGTHS AND LIMITATIONS OF THIS STUDY**

- Recruiting a large sample of patients with healed diabetic foot ulcers (DFUs) to a randomised controlled trial.
- Using blinded assessors to collect primary outcome (total ulcer-free days with limbs intact).
- Multi-modal delivery options for reducing the impact of DFUs intervention sessions.
- Patients are only eligible if they have a good command of the English language; this may limit generalisability of the results of the trial.

## Introduction

 Diabetes affects approximately one in sixteen adults in the UK.^1^ Foot ulceration is a prevalent and costly complication, impacting up to 25% of individuals with diabetes during their lifetime.^2^ Prognosis remains poor: only 49% of people with an ulcer in England and Wales are ulcer-free (and alive) after 12 weeks of ulceration,^3^ and approximately 85% of major amputations in people with diabetes are preceded by an ulcer on the foot.^4^ Even after healing, recurrence is common, with 40% of people re-ulcerating within a year.^5^ The burden extends beyond physical health—32% of patients experience depression, which triples mortality risk.^6^ In 2014–2015, the National Health Service (NHS) expenditure on ulceration and amputation care was estimated at £837–962 million, representing 0.8%–0.9% of the budget^7^ in England.

Although the National Institute for Health and Care Excellence (NICE), the NHS, and Diabetes UK have identified improved foot care as a priority,^4 7 8^ effective preventive strategies remain limited. Current NICE recommendations emphasise risk assessment, referral and basic education, yet systematic reviews indicate education alone does not improve outcomes.^9^,^15^ Consequently, NICE has called for interventions addressing psychological and behavioural determinants, which are critical to ulcer prevention and healing.^4^

The reducing the impact of diabetic foot ulcers (REDUCE) intervention,^15 16^ developed under Medical Research Council guidance on complex interventions,^17^,^19^ directly targets these factors. It aims to (i) reduce re-ulceration risk among patients with recently healed ulcers and (ii) improve healing if recurrence occurs. REDUCE combines behavioural and psychological strategies, focusing on four core goals: daily foot-checking, rapid self-referral for changes in foot health, graded physical activity when ulcer-free and emotional management. These behaviours are intended to increase ulcer-free days (a measure which encapsulates both healing and recurrence) and improve long-term outcomes. The intervention was informed by systematic reviews, patient input, and theoretical modelling, and its development process is reported separately.^20^ Here, we describe a randomised controlled trial (RCT) of the REDUCE intervention and associated work, including an embedded mixed-methods process evaluation and two additional studies. The first is a bio-mechanistic study looking at the molecular pathways and the second is a methodological retention study. Further detail can be found in the additional files (see onlineadditional files 1  2). A pilot trial^21^ was conducted before this RCT, with the associated publication currently submitted for review.

## Methods and analysis

### Study objectives

To measure the effectiveness and cost-effectiveness of the REDUCE intervention, compared with usual care, for people with healed diabetic foot ulcers (DFUs). A full list of the trial objectives is provided in box 1. The trial also included an embedded process evaluation to establish whether the REDUCE intervention was delivered, received and brought about change in the ways intended by the programme developers.

Box 1 **REDUCE objectives**

**Primary objective**

The primary outcome of this trial is total ulcer-free time with limbs intact (ie, without amputation) between randomisation and the end of follow-up (18 months post-randomisation) as measured in days. The number of days increases if the REDUCE intervention delays the onset of ulceration, accelerates healing, reduces the risk of amputation or improves survival.

**Secondary objectives**

- Clinical outcomes.
  - Whether the patient remained ulcer-free.
  - Time to re-ulceration.
  - Total number of ulcers.
  - Proportion of patients deceased.
  - Time to death.
  - Whether patient had major amputation.
  - Time to major amputation operation.
  - Whether patient had minor amputation operation.
  - Time to minor amputation.
  - Days in hospital related to foot ulcer disease.
  - Days in hospital not related to foot ulcer disease.
- Psychological/behavioural risk factors targeted in REDUCE to examine mechanisms.
- Economic outcomes to examine cost-effectiveness.

REDUCE, reducing the impact of diabetic foot ulcers.

### Study design

A multi-centre, parallel group, superiority RCT with two arms and an embedded process evaluation. The study is single-blinded, with the outcome assessor blinded.

### Study setting

This is a multi-centre RCT involving up to 30 clinical sites recruiting a total of 544 participants. It is anticipated that the participant population will be recruited from specialist multidisciplinary diabetic foot clinics at participating NHS Trusts from across the UK. The intervention will be delivered remotely by healthcare professionals (HCPs) who are independent of the participating Trusts. HCPs will be invited to take part in the process evaluation.

Usual care for both arms of the study will be provided by the clinical care teams in primary care, secondary care and community health services, including community podiatry teams.

Clinical outcome data will be extracted from any relevant healthcare records, including (but not limited to) primary care, secondary care, community health and podiatry records.

### Eligibility criteria

To be included, patients need to fulfil all eligibility criteria, which are presented in box 2. Eligibility will be confirmed by an appropriately delegated person from the recruiting site. Included HCPs need to meet all the eligibility criteria to participate; the criteria can be found in box 3.

Box 2 **REDUCE participant eligibility criteria**

**Participant inclusion criteria**

- Has diabetes (according to WHO criteria).
- Is aged 18 years or over.
- Has two lower limbs (ie, has not had major amputation of either lower limb).
- Has a recently healed diabetic foot ulcer (DFU) (if more than one, all must be healed), defined as fully epithelialised with no drainage, healed for a minimum of two weeks and up to a maximum of twelve weeks.
- Has cognitive capacity to provide informed consent, to engage with the study intervention (as digital and written handbook versions), to take part in interviews (if randomised to the intervention and selected as part of a sub-sample), and to provide follow-up data.
- Has sufficient command of the English language and is able to engage with the intervention and to provide follow-up data.

**Participant exclusion criteria**

- Has active Charcot neuro-osteoarthropathy.
- Presence of active diabetic foot ulceration.
- In the acute phase of a diagnosed mental illness where being approached about participation could be an extra burden (eg, currently under the care of the MH crisis team or admitted to hospital at the time of recruitment).
- Has previously been randomised to the REDUCE pilot trial.
- Has previously been randomised to this REDUCE trial.
- Is currently taking part in another study which would affect the outcomes of this study (eg, DFU wound healing medicinal product trial or other behavioural intervention study).
- Has a healed DFU, defined as fully epithelialised with no drainage, healed for more than twelve weeks.

REDUCE, reducing the impact of diabetic foot ulcers.

Box 3 **REDUCE healthcare professionals’ eligibility criteria**

**Healthcare professionals’ (HCPs) inclusion criteria**

- A HCP involved in the delivery of the REDUCE intervention.
- Willing to take part in the process evaluation (by completing questionnaires, participating in a qualitative interview, and having sessions audio-recorded for fidelity assessment).

**HCPs’ exclusion criteria**

- Unwilling to provide informed consent.

REDUCE, reducing the impact of diabetic foot ulcers.

### Participant recruitment – trial participants

Over a 30-month recruitment period, potential participants will be identified using two main routes. First, by being screened by their clinical care team in specialist multidisciplinary diabetes foot clinics at participating NHS Trusts while attending a clinic appointment. Second, by screening clinical caseloads against the eligibility criteria in the trial sites. Medical records will be accessed only by a member of the existing clinical care team (not by the research team) to establish eligibility.

Those eligible will be approached about the trial by their clinical care team during a scheduled clinic visit and given details about the study, including a participant information sheet (PIS). Patients will be provided with a PIS as soon as possible after healing of all their foot ulcers but will only be recruited and consented at a separate clinic visit after the ulcer has remained healed for a period of at least 2 weeks (ie, to align with the clinical definition of healing). If their foot ulcer breaks down during this 2 week period, potential participants can be re-screened for eligibility into the trial three more times. Potential participants with a healed foot ulcer can be approached about taking part in the trial up to twelve weeks after their foot ulcer is defined as healed.

The PIS will also include details about the optional elements of study, including the qualitative interviews which form part of the process evaluation and, where relevant (eg, in participating sites), the biological mechanisms sub-study, a separate written consent form (see online supplemental material) will be obtained by a member of the local research or clinical team as identified on the delegation log.

The potential participant will be allowed as much time as they wish to consider the information and will be given the opportunity to question the Principal Investigator, the research team, their General Practitioner or other independent parties to decide whether they will participate in the study. Written consent will be obtained by a member of the local research or clinical team as identified on the delegation log.

**Figure 1:**
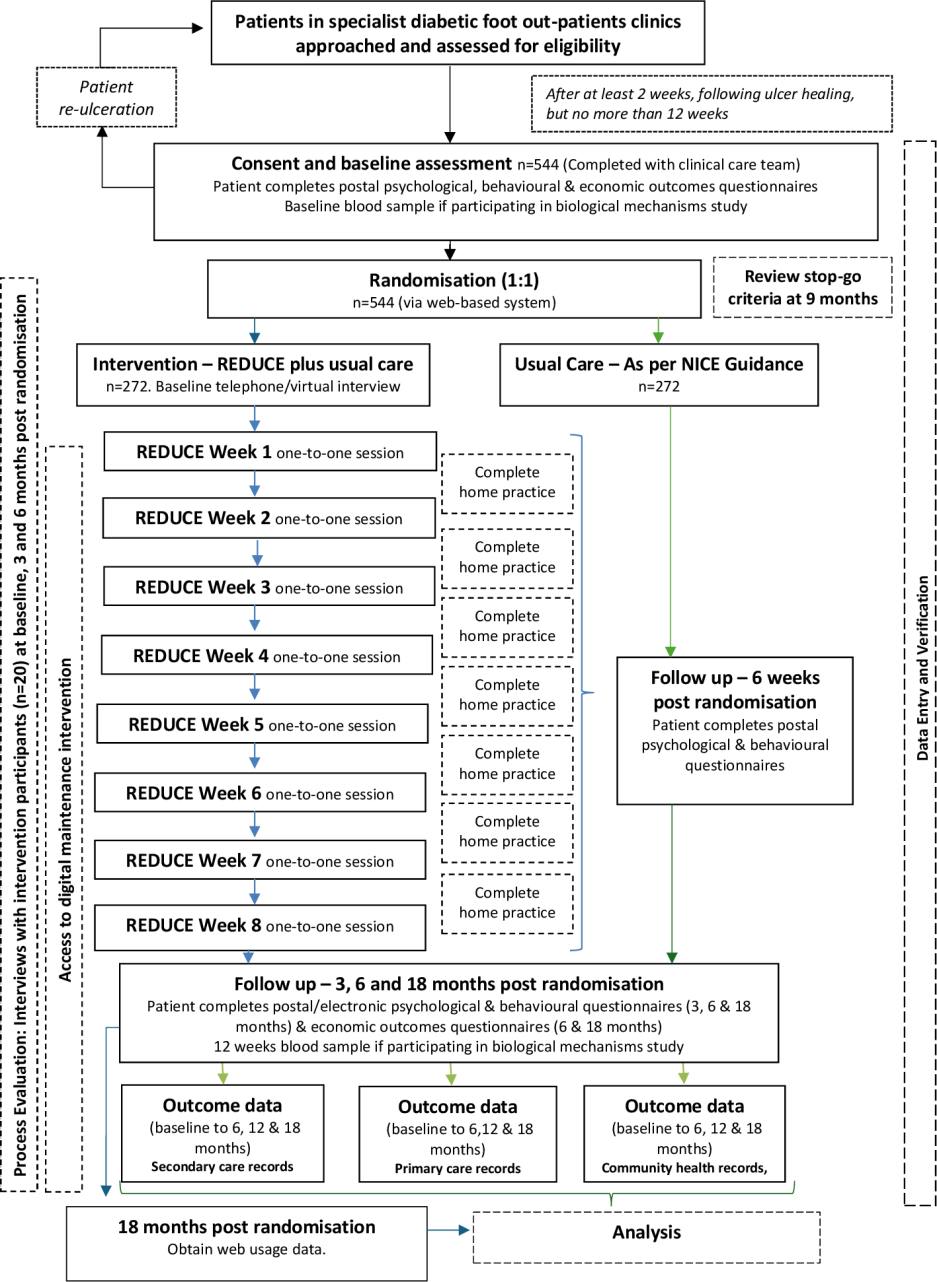
Trial flowchart - participants. NICE, National Institute for Health and Care Excellence; REDUCE, reducing the impact of diabetic foot ulcers.

Figure 1 provides an overview of the participant pathway through the trial.

### Participant recruitment – healthcare professionals

HCPs will be appointed to deliver the REDUCE intervention by the trial Sponsor. HCPs will be provided with an information sheet and consent form. The HCPs’ consent forms cover participation in all relevant aspects of the trial and process evaluation, including questionnaire completion, audio-recording of sessions for fidelity assessment and participation in qualitative interviews.

**Figure 2:**
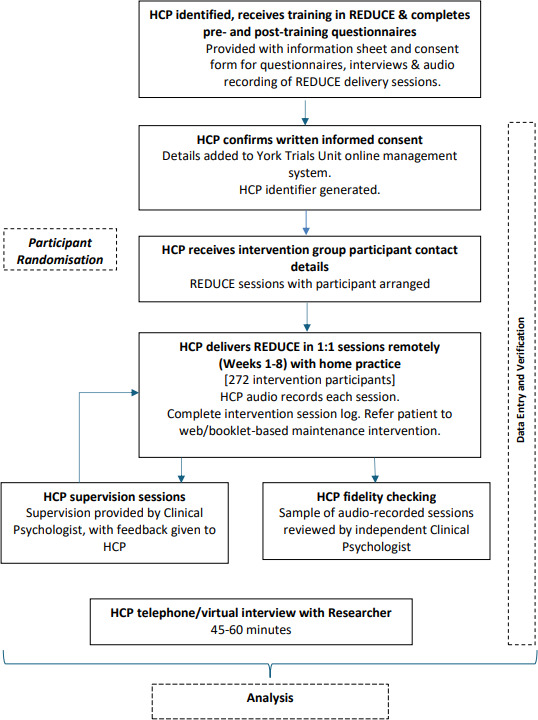
Trial flowchart – healthcare professional participants. HCP, healthcare professional; REDUCE, reducing the impact of diabetic foot ulcers.

Figure 2 provides an overview of the HCP participant pathway through the trial.

### Randomisation

Following consent and completion of all baseline data collection and assessments, trial participants will be randomised. Participants will be told which arm they have been allocated to immediately after randomisation by a member of the unblinded local research team at the recruiting Trust where possible, otherwise they will be notified by a delegated unblinded member of the team as soon as possible.

Participants will be allocated to either:

- ***REDUCE intervention plus usual care (intervention arm)***
  Eight weeks of 1 hour, one-to-one sessions with a HCP trained to deliver the REDUCE intervention. Participants will also be able to access the web- and/or booklet-based maintenance intervention. During the intervention period, participants will continue to receive usual standard care.
- ***Usual care alone (control arm)***
  Participants will receive usual standard care.

The randomisation schedule will be stratified by ulcer history (one previous ulcer vs more than one previous ulcer) and formed of randomly permuted blocks of randomly varying sizes using a 1:1 allocation ratio.

Randomisation will be performed by a remote, centralised, online randomisation service provided by the York Trials Unit (YTU). Telephone backup will be available if required. The allocation sequence will be generated by a statistician not involved in the study.

### Study intervention

The central intervention delivery team at the University of Nottingham (UoN) will liaise with participants randomised to receive the REDUCE intervention to arrange each of their intervention sessions. Participants will receive eight one-to-one sessions (one session per week, where able, over a maximum of 12 weeks) with a REDUCE-trained HCP. The sessions will take place as an online video or teleconference via an NHS approved system. Each session will last approximately 1 hour. Intervention participants will be posted a copy of the initiation and maintenance handbooks and the website log-in details. The website is accessible by mobile phone, tablet and computer. The website is developed and hosted by a commercial company, Global Initiative (GI). For further details, see Vedhara *et al*.^20^ Additionally, intervention participants will also be sent a weekly SMS - text message as a reminder of their upcoming intervention session if the contact details have been provided. To support maintaining behaviour change after completion of the intervention sessions SMS and/or emails will also be sent during follow-up as a reminder about foot care behaviour and using maintenance materials if the contact details have been provided. Where required, intervention participants may be contacted regarding missed appointments and to re-book the intervention session.

Participants allocated to the control arm (usual care) will not receive access to the REDUCE intervention one-to-one sessions or the web- or booklet-based intervention materials but will continue to receive treatment as usual, that is, access to footcare support using the established pathways in their local NHS Trust.

### Blinding

The blinding of participants and clinicians is not possible in this study due to the nature of the intervention. As a result, emergency un-blinding will not be required.

All outcome assessors (those collecting clinical outcome data at sites) will, however, be blinded to the allocation and strategies will be employed to minimise risk of un-blinding. Outcome assessors may be involved in the participants’ usual care, but they will remain blinded to trial allocation; they will not be delivering the intervention. Although the participant’s involvement in the trial will be recorded in the medical notes at the site at which they are recruited, in line with requirements of good clinical practice this will not include the allocation arm, which will only be recorded in the case report forms (CRFs). Participants will be advised regarding with whom they can discuss their allocation, and from whom they should withhold this information. Should the participant inadvertently reveal their allocation to an outcome assessor, or the assessor become un-blinded for any reason, that assessor will no longer continue to assess outcomes for that participant, and the unblinding will be recorded in the outcome assessment CRF at the relevant time (6, 12 or 18 months). Additionally, we will ask assessors to indicate which arm they thought participants were in and why at each of the data collection time points.

The YTU statistician conducting the analyses will not be blinded. The primary analysis will be verified by a second statistician at YTU.

### Data collection

Baseline data collection will be completed using paper questionnaires for both clinician and participant-reported data. Participant follow-up questionnaires will be completed on paper, returned by freepost envelope to the central study team, or electronically, depending on participant preference. Telephone completion of follow-up questionnaires with a member of the research team will be offered if required. Retention strategies will be employed to facilitate the return of questionnaires; this will include reminder letters, SMS and emails (where participants have requested to receive questionnaires electronically). The data collection schedules can be found in table 1 for participants and table 2 for HCPs.

**Table 1:** Schedule of assessments: participants

Time point	Identification*	Enrolment†	Allocation	Intervention delivery‡	Follow-ups				
				Delivery of 8 sessions over 12 weeks	6 weeks	3 months	6 months	12 months	18 months
Enrolment:									
PIS	X								
Eligibility screening		X							
Informed consent		X							
Baseline		X							
Randomisation			X						
Interventions:									
REDUCE plus usual care				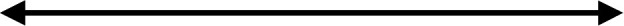					
Usual care only				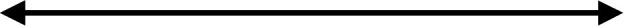					
Assessments:									
Brief IPQ		X			X	X	X		X
CBRQ-SF		X			X	X	X		X
SPANE-P		X			X	X	X		X
PHQ-9		X			X	X	X		X
IPAQ-E-SF		X			X	X	X		X
NAFF		X			X	X	X		X
SPS-5		X			X	X	X		X
ICECAP-A		X					X		X
EQ-5D-5L and EQ VAS		X					X		X
Resource use questionnaire		X					X		X
Participant interviews§		X		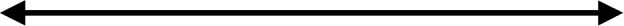		X	X		
Complete home practice§				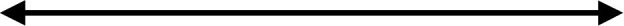					
Session log									
Website access data§					X	X	X	X	X
Clinical outcome data							X	X	X

* Potential participants are identified following the healing of their DFU. This then needs to remain healed for 2 weeks to meet the inclusion criteria.

† Baseline data collected prior to randomisation.

‡ For REDUCE plus usual care participants, 2 weeks have been allocated to allow for intervention sessions to be organised by UoN with the participant. There are eight intervention sessions which can be delivered over 12 weeks. Usual care provision is available to all participants throughout their participation in the trial.

§ Intervention arm only.

CBRQ, Cognitive and Behavioural Responses Questionnaire; EQ VAS, EQ visual analogue scale; ICECAP-A, ICEpop capability measure for adults; IPAQ-E, International Physical Activity Questionnaire – Elderly; IPQ, Illness Perception Questionnaire; NAFF, Nottingham Assessment of Functional Footcare; PHQ-9, Patient Health Questionnaire-9; PIS, participant information sheet; SPANE-P, Scale of Positive and Negative Experience; SPS, Social Provisions Scale.

**Table 2:** Schedule of assessments: healthcare professionals

	Identification	Enrolment*	Preintervention delivery	Intervention delivery	Follow-ups*
**Time point**				*Delivery of 8 sessions over 12* weeks	
Enrolment:					
PIS	X				
Informed consent		X			
Baseline demographics		X			
Assessments:					
Pre-training questionnaire			X		
REDUCE intervention training			X		
Post-training questionnaire			X		
Fidelity assessment – session 4				X	
HCP interview					X

* Either at the end of the allocated intervention delivery or up to when the HCP leaves the intervention delivery team.

HCP, healthcare professional; PIS, participant information sheet; REDUCE, reducing the impact of diabetic foot ulcers.

#### Data collection – clinician completed

Demographic, general health information and clinical data will be collected about trial participants at baseline. Clinical data will include previous medical history (including duration and type of diabetes, most recent HbA1c value, depression, documented peripheral neuropathy, documented peripheral arterial disease, visual impairment, other relevant conditions) and DFU history (including single ulcer vs multiple ulcers, most recent episode confirmed healing date, activity/education/footwear).

The outcome assessors, who are HCPs not involved in the delivery of the REDUCE intervention, will support the collection of clinical outcome data at 6-, 12- and 18-months post randomisation. Data will be collected from relevant healthcare records, to include but not limited to secondary care, GP, community health and community podiatry records. It will include the number of ulcer-free days; days to re-ulceration (if re-ulceration occurs); number of new ulcers; days in hospital (related and not related to foot ulcer disease); adverse events (AEs); serious AEs (SAEs); number of amputations (major and minor) and mortality.

For each scheduled intervention delivery session, the HCPs will collect data on participant attendance, session duration, intervention/modalities delivered and if the participant completed the tasks between sessions. Where a participant does not attend their scheduled session, this will be recorded, and the central intervention delivery team will contact the participant to re-arrange the session.

#### Data collection – participant completed

To assess the psychological and behavioural outcomes, participants will be asked to complete the following validated questionnaires at baseline (prior to randomisation), and at 6 weeks, 3 months, 6 months and 18-months post randomisation:

- Brief Illness Perception Questionnaire.^19^
- Cognitive and Behavioural Responses Questionnaire^18^ – short version – only two items: damage and catastrophising.
- Patient Health Questionnaire-9.^22^
- International Physical Activity Questionnaire – Elderly^23^ – short form.
- Nottingham Assessment of Functional Footcare.^24^
- Scale of Positive and Negative Experience^25^ - positive items only.
- Social Provisions Scale^26^ 5-item scale.

For the health economic analysis, participants will be asked to complete the following questionnaires at baseline, 6 months and 18-months post randomisation:

- ICEpop capability measure for adults (ICECAP-A).^27^
- EQ-5D-5L consisting of the EQ-5D descriptive system and the EQ visual analogue scale.^28^
- Items on health and social care, personal and carer resource use.^29^

All participants will be asked at baseline, 6 weeks, three, six and 18-months about time taken to contact a HCP when last noticing changes in their feet. Participants in the intervention arm will also be asked questions about recent usage of the handbooks (at three, six and 18-months) and about practising what they have learnt during the intervention session period (at 6 weeks and 3 months).

#### Data collection – web-based maintenance intervention

Intervention participants will be able to access the web-based maintenance intervention using an assigned sign-up code that enables the central intervention delivery team at UoN to identify their website accesses. Access and usage data are accessible by the commercial company (GI) via website analytics, for example, Matomo Analytics. Usage data will be collected, which includes the number of visits to the website, pages viewed, duration of views on each page and the device used to access the website. Intervention participants will be able to access the website during the follow-up period of 18 months post-randomisation.

#### Data collection – interviews with selected trial participants (intervention arm)

A sub-sample of approximately 20 participants who are randomised to the REDUCE intervention arm will be interviewed at three time points: at baseline (preintervention) and approximately three and 6 months later. Baseline interviews will explore participants’ perceptions and experiences of managing DFUs pre-trial, and their hopes and expectations of the REDUCE programme and reasons for taking part in the trial. Follow-up interviews will explore participants’ engagement (or lack thereof) with the REDUCE programme’s initiation and maintenance phases, and whether this engagement leads to emotional, psychological and behavioural changes aligned with the programme’s theory (see logic model – online additional file 3). Interviews will be conducted by an experienced qualitative researcher and transcribed in full. For more information, see Hart *et al*.^30^

#### Data collection – interviews with healthcare professionals

HCPs recruited to deliver the intervention will be interviewed near to trial completion (ie, after they have delivered their last intervention session with their last participant). Interviews will explore: their experiences of the REDUCE facilitator training and delivering the one-to-one sessions (including any challenges encountered); their perspectives on the impact of the intervention on different patient-participants; their experiences of supervision and peer support and their views about the resourcing and support needed to rollout and deliver the REDUCE programme in routine clinical care. Interviews will be conducted by an experienced qualitative researcher and transcribed in full. For further information, see Hart *et al*.^31^

#### Data collection – assessing intervention fidelity

All intervention sessions will be audio-recorded. Assessment of intervention fidelity*,* that is*,* was the treatment delivered as intended, will be conducted, and it will focus on the delivery of sessions four and six. Ten per cent of randomly chosen participants, stratified by therapist, HCP profession (if appropriate) and year of trial recruitment, will be rated and 15% of these will be double rated for inter-rater reliability. The tool contains item descriptors and numerical scores. We will report overall integrity as well as component integrity. The selected sessions will be rated using the fidelity tool designed specifically for the REDUCE intervention by two skilled assessors.

### Participant payments

Patient-participants will receive a £5 shopping voucher for completion of the baseline questionnaire. A £5 voucher will be sent with each questionnaire at 6 weeks, 3 months, 6 months and 18-months post-randomisation. A final £5 shopping voucher will be sent to participants for completion of all study questionnaires at the end of the study, as a “thank you”.

### Sample size

As there is limited available data on the number of ulcer-free days on which to base a sample size, our calculation is based on the difference in percentage of participants remaining ulcer-free over follow-up. We acknowledge that it is unconventional that our sample size calculation does not match our primary analysis (ie, using a sample size calculation for proportions when the primary outcome ‘ulcer free days’ is measured as a continuous count variable). However, the approach we have adopted can be regarded as conservative as it will underestimate the power of our analysis.

Approximately 60% of patients will still be ulcer-free at 12 months^32^ (40% within 3 years). Thus, we assume that 55% of control participants will remain ulcer-free over the 18-month follow-up. Our target sample size will permit detection of a 15% increase in patients remaining ulcer-free over follow-up that is, from 55% to 70% (risk ratio=1.3). An intervention effect of this size, with 90% power and 5% significance, requires 217 patients per group (Stata V.15, χ^2^ test comparing two independent proportions). Allowing for 20% loss to follow-up increases the total sample size to 544 (ie, 272 in each group). Experience from previous trials with comparable patient groups suggests that the 20% estimate is not unreasonable. For example, the Heels trial reported 11% loss to follow-up over 6 months and Venus III 12% at 12 months.^18 33^

### Additional studies in the REDUCE trial

Two additional studies are being implemented alongside the trial, the biological mechanisms study and a methodological trial on retention.

#### Biological mechanisms sub-study

Multiple psychological and behavioural factors have been reliably associated with DFU outcomes. This National Institute for Health and Care Research (NIHR) Efficacy and Mechanism Evaluation funded sub-study will use the opportunity of the REDUCE trial to elucidate the molecular pathways through which REDUCE and the psychological and behavioural risk factors it changes affect DFU outcomes. Please refer to Additional File 1 for details of this sub-study.

#### Methods for enhancing retention

Strategies can be included to support the recruitment and retention of participants. However, it is also important to rigorously evaluate these strategies by embedding them into clinical trials;^34 35^ also known as Studies Within A Trial (SWAT). The REDUCE trial will include a SWAT to evaluate whether sending a birthday card improves the retention of participants in trials involving an adult population. The results of the SWAT will be published following the completion of the trial. Please refer to Additional File 2 for details of this SWAT.

### Data management

Information will be held securely on paper and electronically. Security measures include appropriate storage, restricted access, and arrangements for disposal of participants’ personal and clinical details based on University of York destruction guidance. Clinical data collected within the CRFs will be processed by YTU using a licensed, automated, electronic system (Teleform), enabling data entry, checking and validation. Participant data collected within the CRFs will be processed by UoN and 10% will be checked and validated. Specific details regarding the processing of the data will be documented in a study-specific data management plan.

The study will be conducted in accordance with the Data Protection Act 2018. The trial team will ensure that participant anonymity is maintained throughout the study and following completion of the study. Participants will be identified on all study-specific documents (except for the informed consent form and enrolment log) only by the participants’ study-specific identifier. Initials will not be used in the study identifiers for the qualitative or fidelity components of the trial. The Investigator Site File will hold an enrolment log detailing the study-specific identifier alongside the names of all participants enrolled in the study.

All documents will be stored securely with access restricted to study staff and authorised personnel. At the end of the study, following completion of the end of study report, YTU will securely archive all centrally held study-related documentation in the Trial Master File for a minimum of 5 years. At the end of the defined archive period, arrangements for confidential destruction will be made.

FG will act as the custodian of the data generated from the main trial data collection.

### Statistical analysis

The primary and secondary analyses of the quantitative clinical data will be pre-specified in a Statistical Analysis Plan, which will be finalised before the end of follow-up. Analyses will be conducted in Stata V.18 or later. Treatment effects and corresponding 95% CIs will be reported, and statistical significance will be assessed at the 5% level. All outcomes will be reported descriptively at all collected time points. Continuous data will be presented using means and SD or medians and ranges as appropriate, and categorical data will be presented using frequencies and percentages.

The primary analysis will be on an intention-to-treat basis, analysing patients in the groups to which they were randomised. The number of ulcer-free days will be analysed using a mixed-effects Poisson regression model, or negative binomial model as appropriate. The model will adjust for ulcer history and other relevant baseline covariates as fixed effects. Centre will be adjusted for as a random effect. Length of follow-up will be incorporated into the model to consider participants who are lost to follow-up before 18 months post-randomisation. The total number of days spent in hospital will be analysed in a similar manner.

The proportion of participants remaining ulcer-free will be compared between the two groups using a mixed-effect logistic regression including the same fixed and random effects used in the primary analysis. Other binary secondary outcomes will be analysed in a similar manner.

Time to re-ulceration will be analysed via a Cox Proportional Hazards regression model adjusting for the same fixed and random effects used in the primary analysis. Other time-to-event secondary outcomes will be analysed in a similar manner.

Psychological/behavioural outcome measures will be analysed using mixed-effect linear regression models adjusting for relevant baseline covariates as fixed effects and centre as a random effect.

### Health economic analysis

A healthcare service/personal social services perspective will be adopted for the primary analyses; this will be extended to a broader perspective (patient/carer and societal) including lost productivity and personal costs. A resource use measure has been developed using a modified Delphi process involving patients and HCPs who work with people with DFUs.^29^ The intervention and usual care resource use will be calculated based on the intervention sessions logs and interviews with the trial clinical team. Published unit costs will be used to value resource use in £ sterling.^36 37^

Two health economic outcome measures, EQ-5D-5L^38^ and ICECAP-A,^39^ were tested in the REDUCE pilot trial to assess participant completion and missing items. Based on review of the baseline pilot trial data both measures were consistently completed with no missing data. Both measures will be included in the main trial to allow assessment of the benefits on patients’ well-being (ICECAP-A) and health-related quality of life (EQ-5D-5L).

The health economic analyses will be developed using STATA V.18 or later and Excel for Microsoft 365. Discounting will be applied at 3.5% as a base case.^40^

Regression methods will estimate incremental costs and effects, with appropriate baseline adjustment. The impact of missing data will be determined using suitable methods.^41^ The primary analysis will be a cost-utility analysis presenting an incremental cost per quality-adjusted life year gained over 18 months. Sensitivity analyses will include parameter variation in costs and effects and selected scenario analyses. Uncertainty will be explored using bootstrapping, with cost-effectiveness acceptability curves presented. We will estimate the net monetary benefit gained from REDUCE. Appropriate societal willingness to pay thresholds^40^ will be used to determine whether REDUCE could be considered an effective use of NHS resources. Additional analyses will present the cost per year of sufficient capability based on the ICECAP-A. An incremental cost per ulcer free days gained at 18 months will be calculated based on the primary trial endpoint with deterministic sensitivity analysis to examine the effect of parameter variation. Other outcomes will be presented as part of a cost-consequence analysis. Using the trial data, supplemented by best available evidence and informed by good practice,^42^ we will estimate the likely impact of using the REDUCE intervention on UK NHS budgets.

The potential for economic modelling to extrapolate longer-term cost-effectiveness beyond the trial period will be assessed on the clinical and cost effectiveness of REDUCE at 18 months (based on statistical significance, range of CIs and clinically important differences over time), availability and plausibility of data inputs identified from a structured literature review and expert elicitation, and where necessary, plausibility of assumptions. The conditions to inform the model development will be detailed in the health economic analysis plan. The decision to develop the economic model will be made with the Programme Management Group and the joint independent Trial Steering and Programme Steering Committee (TS-PSC).

### Qualitative data analysis

Interviews with patient-participants and HCPs will be analysed using thematic and framework approaches. To maximise rigour and conform to qualitative data reporting standards (eg, the Consolidated criteria for Reporting Qualitative research),^43^ at least two experienced qualitative researchers will be involved in coding, identification and refinement of inductively and deductively identified themes. The qualitative software package NVivo will be used to support data coding and retrieval.

### Patient and public involvement

Patients and carers have been involved in the design of the trial and will be involved in the management of the research, analysis of results and the dissemination of findings. The PIS was co-developed with the patient and public involvement (PPI) group. In addition, members of the PPI group have provided feedback on the content of participant-facing study documents, the digital and handbook versions of the maintenance intervention and have tested the feasibility and duration for completing the psychological, behavioural and health economic questionnaires. The advice of this group will be sought throughout the course of the trial if there have been changes to participant-facing documents during the course of the trial. The group will also be consulted on the interpretation of the findings of this trial and how best to disseminate these.

### Protocol amendments

Any protocol amendments will be approved by the Sponsor (UHDB) and the Funder (NIHR PGfAR) prior to submission to the REC and the Health Research Authority. Documentation will be provided to study sites for their local review and implementation as required.

### End of study

The end of study will be defined as ‘last participant last visit’ that is, the date at which the last participant has completed their final study process. The CI will notify the Sponsor, participating sites and REC within 90 days of the end of study.

## Ethics and dissemination

### Ethics approval

Research Ethics approval was granted by Wales 3 Research Ethics Committee (REC reference 22/WA/0053) on 16 March 2022.

### Safety reporting

Non-serious adverse events (NSAE) are defined as any untoward medical occurrence in a trial participant (ie, any unfavourable and unintended sign, symptom or disease), which is related to study ulcer and/or to the study treatments (intervention or control). NSAEs which might be expected among DFU participants include re-ulceration, new ulceration and ulcer infection. NSAEs which may be expected do not need to be reported as part of the trial; however, sites will need to follow their usual local reporting procedures.

SAEs are defined as any untoward medical occurrence that:

1. Results in death.
2. Is life-threatening.
3. Requires inpatient hospitalisation or prolongation of existing hospitalisation.
4. Results in persistent or significant disability/incapacity.
5. Consists of a congenital anomaly or birth defect.

Other ‘important medical events’ may also be considered serious if they jeopardise the participant or require an intervention to prevent one of the above consequences. The term “life-threatening” in the definition of “serious” refers to an event in which the participant was at risk of death at the time of the event; it does not refer to an event which hypothetically might have caused death if it were more severe.

Causality and expectedness of the SAE will be confirmed by the Chief Investigator or another clinical member of the Trial Delivery Group (TDG) if the CI is unavailable. SAEs that are deemed to be unexpected and related to the trial will be notified to the Research Ethics Committee (REC) and Sponsor within 15 days. All such events will be reported to the TS-PSC at their next meeting.

All events will be followed up until the event resolves or a decision is made for no further follow up. Participants experiencing SAEs which are deemed to be related to the trial treatments (intervention or control) and which remain ongoing at the time of participant trial exit will be followed up for one further month beyond trial exit.

Where repeated AEs (serious or non-serious) of a similar type are observed, these will be discussed with the TDG and other relevant groups and will be onward reported to the REC and Sponsor should concerns be raised in relation to the type of event and/or frequency observed.

### Withdrawal

Participants will have the right to withdraw from the study at any time, without giving a reason. In addition, the Investigator may advise that a participant be discontinued from the study at any time if the Investigator considers it necessary for any reason. However, the decision on full withdrawal will remain with the participant at all times. It will be made clear in the PIS that should they wish to withdraw, this will not affect their future clinical care, although data collected to that point as part of the research will be retained.

HCPs will have the right to withdraw from the questionnaires and interviews at any time without giving a reason. Any study data provided up to that point will still be used and HCPs will be notified of this prior to consent.

### Trial monitoring

No formal monitoring visits will be planned for this study unless concerns arise regarding participant safety or trial conduct. A monitoring plan will however be generated for the study to outline the range of centralised monitoring activities (eg, eligibility, consent and safety checks) which will be undertaken in this study. Central monitoring of consent and participant eligibility will be completed for 100% of participants alongside routine validation of all data collected during data processing. Annual audit of site files will occur for each participating site using self-completed checklists.

TDG and TS-PSC meetings will be held to review trial conduct including all data monitoring and ethics issues.

### Management committees

#### Trial delivery group (TDG)

The day-to-day running of the work described in this protocol will be overseen by the TDG which consists of Co-Chief Investigators, Programme Manager, Trial Manager, Trial Statistician and key members of the team from UoN, YTU, Kings College London, Cardiff, Southampton and Edinburgh. This group will meet monthly during set-up and move to bi-monthly online meetings once in follow-up. Any problems with study conduct will be raised and addressed during TDG meetings.

#### Joint trial steering and independent programme steering committee (TS-PSC)

The TS-PSC will oversee and supervise the progress of the trial and ensure that it is being conducted according to the protocol and the applicable regulations. The TS-PSC is an independent body that includes members who are not involved with the running of the trial. The TS-PSC consists of five members: an independent Chair with expertise in health psychology, an independent clinician with expertise in diabetes, an independent member with expertise in podiatry, an independent statistician and an independent PPI representative. Representatives from the TDG will attend the TS-PSC to provide the updates, to include Co-Chief Investigators, Trial Manager, Trial Statistician, Programme Manager, Sponsor representative(s) and a Funder representative. Other members will be invited on an ‘as required’ basis. A separate Data Monitoring and Ethics Committee will not be convened for this trial. The TS-PSC will be responsible for all data monitoring and ethics issues raised throughout the trial. The TS-PSC will meet bi-annually over the duration of the trial.

#### Programme management group (PMG)

The study is part of a larger programme of work, which will be overseen by the REDUCE PMG. The PMG consists of all the applicants and collaborators on the wider programme (including members of the TDG) and one lay/PPI member. The PMG will meet biannually over the programme to oversee the management of all work packages, including the work described here. The PMG will be notified of any problems with study conduct. A representative from the sponsoring organisation will also attend these meetings, where able/required.

### Dissemination plans

The results from this trial will be submitted for publication in appropriate peer-reviewed journals, regardless of the size or direction of the observed effects. A publication policy will be produced to detail authorship, acknowledgements and the review process for the main publications arising from the research. They will also be presented at national and international conferences for both academic and clinical colleagues. A plain language summary of the results will be co-produced with the PPI group and made available to participants who have expressed an interest in receiving this information. The PPI group will be encouraged to participate in the dissemination of the results to ensure patient accessibility.

The Standard Protocol Items: Recommendations for Interventional Trials reporting guidelines for reporting the REDUCE protocol.^44^

#### Trial status

Recruitment started on 8 July 2022 and was completed on 27 September 2024. Follow-up is expected to be completed by 31 March 2026. End of study is 31 May 2027.

## Supplementary material

10.1136/bmjopen-2026-118771 online supplemental file 1

10.1136/bmjopen-2026-118771 online supplemental file 2

10.1136/bmjopen-2026-118771 online supplemental file 3

10.1136/bmjopen-2026-118771 online supplemental file 4
