# Supplementary material for: Reducing the impact of diabetic foot ulcers (REDUCE): study protocol for an effectiveness and cost-effectiveness randomised controlled trial with embedded process evaluation
Source: BMJ Open. 2026 May 24;16(5):e118771. doi: 10.1136/bmjopen-2026-118771 (PMC13202030; doi:10.1136/bmjopen-2026-118771)
Supplement: online supplemental file 1 [file bmjopen-16-5-s001.pdf]

PARTICIPANT CONSENT FORM
(Version 1.1; 05 April 2022)

Title of Study: REDUCE Trial: Reducing the impact of diabetic foot ulcers

Participant ID: | | | | | | |

Please initial each box

1. I confirm that I have read and understand the information sheet version number XX dated XXXX for the above study. I have had the opportunity to ask questions about the study and any questions have been answered to my satisfaction.

2. I understand that my participation is voluntary and that I am free to withdraw at any time, without giving any reason, and without my medical care or legal rights being affected. I understand that should I withdraw then the information collected so far cannot be erased and that this information may still be used in the project analysis.

3. I understand that relevant sections of my medical notes and data collected in the study may be looked at by authorised individuals from the University Hospitals of Derby & Burton NHS Foundation Trust, the research group (including the University of Nottingham, York Trials Unit, the University of Edinburgh, King's College London, Swansea University, University of Southampton, Cardiff University, the University of Bristol and the University of Manchester) and regulatory authorities where it is relevant to my taking part in this study. I give permission for these individuals to have access to these records.

4. I agree to the storage, including electronic, of personal information for the purposes of the study by [INSERT SITE NAME] NHS Hospital, the Sponsor, University of Edinburgh and York Trials Unit. I understand that any information that could identify me will be kept strictly confidential and that no personal information will be included in the study report or other publication.

5. I agree to University Hospitals of Derby & Burton NHS Foundation Trust, University of Nottingham and York Trials Unit holding copies of my consent form, other study related documents and my contact details to allow them to contact me, including to complete questionnaires. I am happy for authorised individuals at these sites to contact me or my healthcare providers to obtain my current contact details should these change.

6. I understand my GP will be notified of my participation in this study.

7. I understand that the information collected about me may be used to support other research in the future, and may be shared anonymously with other researchers. This will not include any audio recordings, or transcripts of interviews.

8. I understand that after this study has finished, the data collected from me will be securely stored in accordance with requirements of the law after which arrangements for confidential destruction will take place. Information that could identify me will be kept strictly confidential and no personal information will be included in the study report or other publication.

9. I understand that if I am randomly selected to receive the REDUCE package, I will take part in REDUCE. I understand the REDUCE sessions will be audio recorded The REDUCE session recordings will only be used to improve the sessions provided.

PLEASE TURN OVER

Please initial each box

10. **I agree to take part in the REDUCE study**.

In addition to the above statements, **please initial the appropriate box** to indicate whether you also agree to the options below. Your participation in this research study will not be affected if you do not agree to these options.

	**Yes**	**No**
11. I understand that if I am randomly selected to receive the REDUCE package, I may be invited to be interviewed up to three times. **I consent to taking part in the interview(s) if invited.**		
12. I understand the interview(s) will be audio recorded and the interviews transcribed. I consent to audio recordings.		
13. I understand that anonymous direct quotes from the interviews may be used in the study reports and publications. I consent to quotes being used.		
14. I consent to my contact information being retained so that I can be contacted in the future regarding this or related research.		
15. I would like to receive a copy of the study results.		

Name of participant (please print)	Date DD/MM/YYYY	Signature
Name of person taking consent (please print)	Date DD/MM/YYYY	Signature

3 copies: 1 for participant, 1 for the medical notes, original to be kept in the investigator site file.
Additional scanned copy to York Trials Unit.

This study is funded by the National Institute for Health and Care Research (NIHR) Programme Grants for Applied Research (project reference RP-PG-0618-20001)
