# Supplementary material for: Reducing the impact of diabetic foot ulcers (REDUCE): study protocol for an effectiveness and cost-effectiveness randomised controlled trial with embedded process evaluation
Source: BMJ Open. 2026 May 24;16(5):e118771. doi: 10.1136/bmjopen-2026-118771 (PMC13202030; doi:10.1136/bmjopen-2026-118771)
Supplement: online supplemental file 2 [file bmjopen-16-5-s002.pdf]

REDUCE - Biological Mechanisms Sub-study

Background

A mechanistic sub-study is embedded within the REDUCE trial to elucidate the molecular pathways through which REDUCE, and the psychological and behavioural risk factors it changes, affect diabetic foot ulcer outcomes.

Epithelial tissue repair and inflammatory responses are critical processes for the prevention and healing of chronic wounds. Both are impaired in people living with diabetes (1), in part due to dysregulation of circulating immune cells and proteins, including: prolonged production of pro-inflammatory cytokines and diminished type 1 interferon responses observed in diabetic wounds (2,3).

Genes encoding these immune mediators have been previously shown to be sensitive to psychological factors (4). In particular, chronic stress is associated with a characteristic pattern of gene expression known as the Conserved Transcriptional Response to Adversity (CTRA), characterised by upregulation of pro-inflammatory and downregulation of type I interferon–related gene expression (5).

The mechanistic sub-study will test the hypothesis that REDUCE impacts modifies this CTRA pathway, leading to downstream changes in circulating pro-inflammatory cytokines and type 1 interferon responses, and ultimately influencing diabetic foot ulcer (DFU) outcomes.

Objectives

1. To quantify changes in CTRA gene transcriptional responses among patients in both arms of the REDUCE trial – to examine whether REDUCE influences CTRA gene expression.
2. To measure levels of healing-related cytokines and proteins in both arms of the REDUCE trial - to investigate if REDUCE affects the circulating immune environment.
3. To conduct analyses exploring the relative contribution of biological, psychological and behavioural pathways on ulcer outcomes, among patients participating in the REDUCE trial.

Eligibility criteria

Biological mechanism sub-study inclusion criteria:

- Participating in the main REDUCE trial

Biological mechanism sub-study exclusion criteria

- Unwilling to provide informed consent and undergo venepuncture.
- Venepuncture contraindicated.
- Recruited at sites not participating in the biological mechanisms sub-study (e.g., site does not have the personnel required for phlebotomy).

Participant recruitment

Patients who are identified for the main REDUCE trial at recruiting sites supporting the biological mechanisms study will also be approached about taking part in this additional study. They will be provided with an additional PIS about the sub-study and be required to complete a separate consent form.

Data collection

Non-fasting peripheral blood samples (approximately 15-20ml total) will be taken via venepuncture at baseline and 12 weeks post-randomisation. Research/clinical staff (e.g., nurse, phlebotomist or other suitably qualified person) will take baseline samples in diabetic foot clinics, or other appropriate settings (e.g., hospital phlebotomy clinic, GP surgery), after participants are randomised in the main REDUCE trial, but prior to receiving any REDUCE intervention sessions. The follow-up 12-week sample will be collected as part of an additional study visit. Samples will be analysed according to standard protocols for RNA CTRA analysis (5) and an automated multiplex enzyme-linked immunoassay (6) panel of six pro-inflammatory and pro-healing/regulatory cytokines previously linked to healing and non-healing wounds (7).

Participant payments

For those participants involved in the biological mechanisms sub-study, reasonable travel expenses will be covered for additional trips to appropriate locations where blood sampling is taking place. This is in addition to the participant payments for the main REDUCE trial.

Sample size

No previous studies have examined the effect of a psychological and behavioural intervention on CTRA gene responses in patients with diabetes. However, previous research has examined CTRA gene responses to a cognitive-behavioural intervention in patients without diabetes, demonstrating a between group effect size of d=0.7 on a single composite measure combining the 53 CTRA gene-set (5).

Using G*Power 3.1, we calculated that using the same outcome to reliably detect a more conservative effect size of d=0.5, with 80% power, in a two tailed independent samples t-test would require a total sample size of 128 participants (64 per arm). Allowing for 10% attrition, we initially proposed a target sample size of 142 patients (71 per arm). However, recruitment into the sub-study was hindered by delays in approvals and study set-up. Consequently, the target sample size was revised to n=100 participants. This revised sample size is estimated to detect an effect size of d=0.6, with 80% power, in a two tailed independent samples t-test, allowing for 10% attrition.

Data analysis

Analyses will be primarily based on comparisons of the randomly allocated groups, with treatment effects estimated using appropriate regression models and presented with 95% confidence intervals and two-tailed p-values. The primary analysis will examine between group differences in CTRA responses and cytokine levels at 12 weeks using an analysis of covariance (ANCOVA), comparing composite CTRA response scores at 12 weeks, with baseline CTRA response scores included as a covariate. Additional analyses will explore the evidence for a treatment effect on the trial primary outcome of ulcer free days with limbs intact, being mediated at least in part by a treatment effect on CTRA response scores.

Funding

This work was supported by the National Institute for Health and Care Research (NIHR) under its Programme Grant for Applied Research (PGfAR) [Reference: RP-PG-0618-20001] and NIHR Efficacy and Mechanism Evaluation (EME) [Reference: NIHR154807].

References

1. Blakytny R, Jude E. The molecular biology of chronic wounds and delayed healing in diabetes. Diabet Med J Br Diabet Assoc. 2006 June;23(6):594–608.

2. Tarnuzzer RW, Schultz GS. Biochemical analysis of acute and chronic wound environments. Wound Repair Regen Off Publ Wound Heal Soc Eur Tissue Repair Soc. 1996 Sept;4(3):321–5.

3. Wolf SJ, Audu CO, Joshi A, denDekker A, Melvin WJ, Davis FM, et al. IFN-κ is critical for normal wound repair and is decreased in diabetic wounds. JCI Insight. 7(9):e152765.

4. Cole SW. The Conserved Transcriptional Response to Adversity. Curr Opin Behav Sci. 2019 Aug;28:31–7.

5. Antoni MH, Lutgendorf SK, Blomberg B, Carver CS, Lechner S, Diaz A, et al. Cognitive-behavioral stress management reverses anxiety-related leukocyte transcriptional dynamics. Biol Psychiatry. 2012 Feb 15;71(4):366–72.

6. Dysinger M, Marusov G, Fraser S. Quantitative analysis of four protein biomarkers: An automated microfluidic cartridge-based method and its comparison to colorimetric ELISA. J Immunol Methods. 2017 Dec;451:1–10.

7. Patel S, Maheshwari A, Chandra A. Biomarkers for wound healing and their evaluation. J Wound Care. 2016 Jan 2;25(1):46–55.
