# Supplementary material for: Reducing the impact of diabetic foot ulcers (REDUCE): study protocol for an effectiveness and cost-effectiveness randomised controlled trial with embedded process evaluation
Source: BMJ Open. 2026 May 24;16(5):e118771. doi: 10.1136/bmjopen-2026-118771 (PMC13202030; doi:10.1136/bmjopen-2026-118771)
Supplement: online supplemental file 3 [file bmjopen-16-5-s003.pdf]

Additional File 2: Birthday Card SWAT Protocol

Title: Effect of sending a birthday card on the retention of participants in an adult healed diabetic foot ulcer trial

Objective of this SWAT
To evaluate whether sending a birthday card improves the retention of participants in trials involving an adult population (aged 18 and above).

Study area: Retention, Follow-up
Sample type: Participants

Background
Randomised controlled trials are regarded as the gold standard for evaluating healthcare interventions. However, trialists often experience poor participant retention (i.e., poor questionnaire responses) and these have serious consequences on the validity, reliability, and generalisability of study results [1],[2]. Hence, it is important to rigorously evaluate participant recruitment and retention strategies to produce evidence on effective (and ineffective) strategies to help trialists make better-informed decisions in conducting trials [3]. The REDUCE trial aims to include a SWAT to evaluate the effectiveness of sending a birthday card on participant retention in a diverse adult population. A number of studies have explored the use of non-monetary incentives (e.g., providing pens or using Post-it notes to encourage retention) [3],[4]; this SWAT would further contribute to the existing body of research that aims to enhance participant retention in trials. Moreover, the inclusion of more participants as a result of this SWAT gives it the potential to be incorporated into a meta-analysis with similar studies, such as SWAT 79 [5].

Interventions and comparators
Intervention 1: A birthday card for the whole of the trial (i.e., if a participant has two birthdays during their 18-month follow-up, they will receive birthday cards twice).
Intervention 2: No birthday card

Index type: Birthday cards, follow-up

Method for allocating to intervention or comparator
All participants recruited into the host trial will be eligible to take part in this SWAT. At the point of recruitment to the main trial, participants will be randomly allocated 1:1 to either receive birthday cards throughout the trial (i.e., until the participant exits the trial at final follow-up or fully withdraws) or not to receive birthday cards throughout the trial. Block randomisation will be stratified by the main trial allocation using randomly-permuted blocks of randomly-varying sizes. The allocation sequence will be generated by the trial statistician who is not involved in the follow-up of participants.

Outcome Measures
The primary outcome of this SWAT is whether the questionnaire was returned at the first-time point following receipt of the first birthday card. Secondary outcomes include:

1. Whether the questionnaire was returned at the first-time point following receipt of the second birthday card.
2. Response rate at each timepoint (6-weeks, 3 months, 6 months and 18 months).
3. Time to response (number of days from date due to date returned) at the first-time point following receipt of the first birthday card.

4. Time to response (number of days from date due to date returned) at the first-time point following receipt of the second birthday card (where applicable).
5. Needed an initial reminder at the first-time point following receipt of the first birthday card.
6. Needed an initial reminder at the first-time point following receipt of the second birthday card (where applicable).
7. Needed a telephone reminder at the first-time point following receipt of the first birthday card.
8. Needed a telephone reminder at the first-time point following receipt of the second birthday card (where applicable).
9. Cost per participant retained at the first-time point following receipt of the first birthday card.
10. Cost per participant retained at the first-time point following receipt of the second birthday card (where applicable).

Analysis Plans

The sample size for this embedded trial will be constrained to the number of participants recruited into the host trial. All participants recruited into the host trial and who are currently participating in the study, at the point at which their birthday card is due to be sent out, will be eligible to take part in this embedded trial.

Analyses will follow the principles of intention to treat, including all participants in the groups they were originally allocated. The primary outcome will be analysed using mixed-effect logistic regression including SWAT allocation, main trial allocation, age, and gender as fixed effects. Recruitment site will be adjusted for as a random effect. Binary secondary outcomes will be analysed in a similar manner. Time to response will be compared between the groups using the frailty extension of the Cox proportional hazards regression including SWAT allocation, main trial allocation, age and gender as fixed effects and recruitment site as a random effect. Subgroup analyses for age and gender will be undertaken for the primary outcome via the addition of an interaction term between the relevant factor and SWAT allocation.

Possible problems in implementing this SWAT

The major challenge associated with the study may be the staff time required to administer this SWAT. Hence, the costs associated with the SWAT will relate to 1) designing, printing, & posting the birthday card (and associated stationary); and 2) staff time for randomisation and administering these activities [5]. Additionally, as the host trial serves a diverse population, some groups – such as Jehovah’s Witnesses that do not celebrate birthdays [6] – may be averse to receiving birthday cards; and care must also be taken to create a birthday card which is not offensive to participants. Therefore, we will liaise with the recruiting site teams to help identify anyone who may be offended to participate in this SWAT or any participant feedback about receiving a birthday card.

References

[1] L. Cureton et al., ‘Randomised study within a trial (SWAT) to evaluate personalised versus standard text message prompts for increasing trial participant response to postal questionnaires (PROMPTS)’, *Trials*, vol. 22, no. 1, p. 502, Jul. 2021.

[2] K. Gillies et al., 'Strategies to improve retention in randomised trials', *Cochrane Database Syst. Rev.*, vol. 3, no. 3, p. MR000032, Mar. 2021.
[3] S. James et al., 'Including a pen and/or cover letter, containing social incentive text, had no effect on questionnaire response rate: a factorial randomised controlled Study within a Trial', *F1000Res.*, vol. 9, p. 623, Jun. 2020.
[4] S. Rodgers et al., 'A study update newsletter or Post-it® note did not increase postal questionnaire response rates in a falls prevention trial: an embedded randomised factorial trial', *F1000Res.*, vol. 7, p. 1083, Jul. 2018.
[5] Backhouse M, Torgerson DJ, Parker A, Cockayne S, 'SWAT 79: Effect of a birthday card on retention and data completion rates in trials involving children'. 2018[Online]. Availablehttps://www.qub.ac.uk/sites/TheNorthernIrelandNetworkforTrialsMethodologyResearch/FileStore/Filetoupload,1359431,en.pdf.
[6] B. Pavlikova and J. P. van Dijk, 'Jehovah's Witnesses and Their Compliance with Regulations on Smoking and Blood Treatment', *Int. J. Environ. Res. Public Health*, vol. 19, no. 1, Dec. 2021[Online]. Availablehttp://dx.doi.org/10.3390/ijerph19010387.
