# Supplementary material for: Reducing the impact of diabetic foot ulcers (REDUCE): study protocol for an effectiveness and cost-effectiveness randomised controlled trial with embedded process evaluation
Source: BMJ Open. 2026 May 24;16(5):e118771. doi: 10.1136/bmjopen-2026-118771 (PMC13202030; doi:10.1136/bmjopen-2026-118771)

Additional file 3: Revised logic model elaborating on intervention techniques deployed in the initiation and maintenance phases of REDUCE

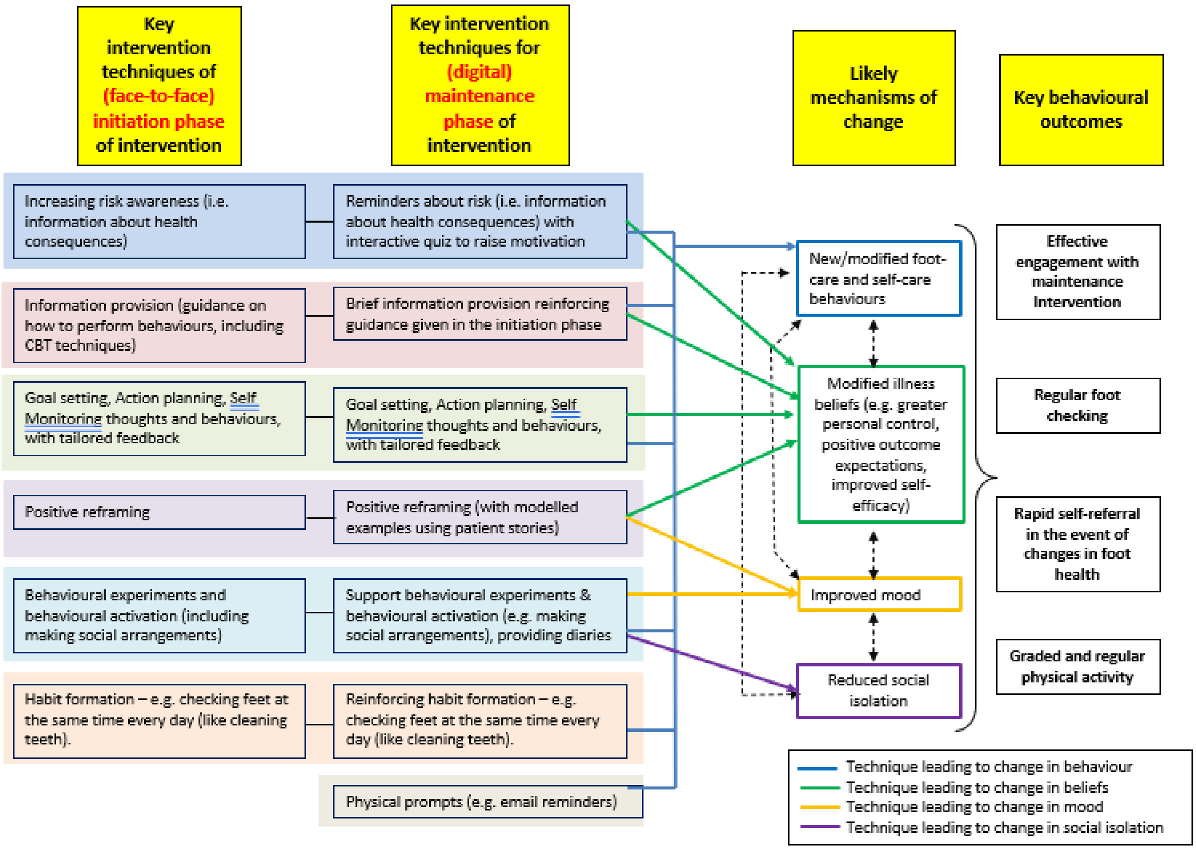

Supplement: online supplemental file 4 [file bmjopen-16-5-s004.pdf]
